# Multi-professional healthcare teams, medical dominance, and institutional epistemic injustice

**DOI:** 10.1007/s11019-025-10252-z

**Published:** 2025-01-23

**Authors:** Anke Bueter, Saana Jukola

**Affiliations:** 1 Department of Philosophy and History of Ideas, Aarhus University, Aarhus, Denmark; 2 Section Philosophy, University of Twente, Enschede, The Netherlands

**Keywords:** Multi-professional teams, Epistemic injustice, Medical dominance, Philosophy of medicine

## Abstract

Multi-professional teams have become increasingly common in healthcare. Collaboration within such teams aims to enable knowledge amalgamation across specializations and to thereby improve standards of care for patients with complex health issues. However, multi-professional teamwork comes with certain challenges, as it requires successful communication across disciplinary and professional frameworks. In addition, work in multi-professional teams is often characterized by medical dominance, i.e., the perspective of physicians is prioritized over those of nurses, social workers, or other professionals. We argue that medical dominance in multi-professional teams can lead to institutional epistemic injustice, which affects both providers and patients negatively. Firstly, it codifies and promotes a systematic and unfair credibility deflation of the perspectives of professionals other than physicians. Secondly, it indirectly promotes epistemic injustice towards patients via leading to institutional opacity; i.e., via creating an intransparent system of credibility norms that is difficult to navigate. To overcome these problems, multi-professional teamwork requires institutional settings that promote epistemic equity of team members.

## Introduction

Multi-professional teams (i.e., teams consisting of professionals from different fields such as medicine, nursing, occupational therapy, and social work) have become common in healthcare institutions (Gadolin and Wikström 2016; see, e.g., Ellis and Sevdalis 2019 on different definitions of multi-professionality). One reason for this is that in aging societies, the number of patients with chronic diseases, complex health issues and comorbidities requiring a multilateral approach has increased (e.g., Ellis and Sevdalis 2019; Pearson-Stuttard et al. 2019). Another reason is the growing degree of specialization of skills and knowledge within medicine and healthcare. Especially for patients with multiple health issues, this results in a need to interact with various professionals. However, even patients with a singular health problem will often require coordinated care by, e.g., surgeons, anesthetists, nurses, physiotherapists, and occupational therapists. Multi-professional collaboration is believed to enable knowledge amalgamation, improve decision-making and contribute to patient safety and treatment outcomes (Babiker et al. 2014). Well-functioning teams can diminish the risk of delayed diagnosis or misdiagnosis, promote the detection of adverse treatment effects, and avoid leaving patients alone in central aspects of managing their conditions. Ideally, multi-professional teamwork thus improves healthcare both epistemically by sharing knowledge and ethically by extending and integrating dimensions of care.

While this sounds very promising, multi-professional teamwork is not without its problems. It can be challenging for both professionals and patients. For instance, such teamwork involves risks of medical errors resulting from communication issues, or of waste of resources due to inefficient collaboration (Babiker et al. 2014). The very teamwork that ideally leads to epistemic and ethical improvements in care also creates additional tasks for healthcare professionals concerning the integration of multiple perspectives. In addition, it can be difficult for patients to navigate such complex healthcare environments. Healthcare systems have traditionally been ‘siloed’ by specialties, and overcoming this fragmentation requires effort especially from patients with complex needs (e.g., Lynch et al. 2022). While the involvement of multiple professionals may be necessary to address patient needs in a comprehensive manner, it threatens to impose high cognitive, organizational, and communicative demands on patients. In particular, this will be the case where multi-professional treatment conflicts with the aim of *continuity of care*.

From a patient perspective on navigating the healthcare system, continuity of care has been described in terms of a continuity of contacts as well as of continuity across sectors and professional disciplines (Folker et al. 2019; cf. also Gulliford et al. 2006). Continuity of contacts refers to a patient preference for stability in contact persons within different domains, such that they can build stable trust relations to particular professionals and do not need to recount their story to someone new again and again. In addition, continuity of care would require that patients receive coordinated care, in the sense that they don’t have to be the communicative link between different professionals themselves. This is also a prerequisite for coherent care, i.e., avoiding conflicting prescriptions and measures.^1^ When patients are in contact with professionals from different specialties, disruptions in the flow of information are more common. This is demonstrated by the findings of a study on the experiences of patients with long-term mental health issues:

“[W]hile some participants experienced coherent and coordinated care within a specific sector or discipline, this tended to end abruptly when they crossed sectors and professional disciplines, or when decisions made in one sector (e.g., over medication and treatment regime) influenced possibilities to comply with decisions made in other sectors and disciplines (e.g., concerning employment activities, social skills training, or eligibility for social support)” (Folker et al. 2019, 1921).

The respective continuity of care has been identified as both a challenge and an aim in the care for complex patients. It is an aim as it improves outcomes and quality of life among people with, e.g., severe mental disorders (Folker et al. 2019) or chronic somatic diseases such as diabetes or asthma (Cabana et al. 2004). It is challenging to implement because it requires a variety of institutional structures to enable it. For instance, the Danish mental healthcare system has implemented several projects in the last 15 years to improve continuity of care for people with severe mental disorders as well as dual disorders, i.e. concurrent mental health and substance use disorders. A comprehensive analysis of these projects found major barriers to continuity of care in four different areas: (a) lack of models for collaboration, (b) differences in professional cultures and methods, (c) lack of shared channels of communication, and (d) intersectoral differences in management, economy, and legislation (Kristensen et al. 2019).

In this paper, we are primarily concerned with differences in professional cultures and methods (b) (even though these will of course affect, and be impacted by, factors within the other three dimensions). Kristensen et al. (2019) remark that difficulties in this area stem from deeply rooted differences in terminology, objectives, and methodology. We will expand on this point by arguing that different professions often diverge regarding underlying conceptual and metaphysical, epistemological, and even ethical perspectives. While this can, in principle, promote a fruitful pluralism, it can also be complicated by asymmetric power relations, which lead to *institutional epistemic injustice*.

The traditional picture of multi-professional teamwork in healthcare has been hierarchical (Willis 1983; Tousijn 2012): Medical doctors have been leading other professionals, e.g. nurses and physiotherapists, who have more of an assisting role. Contrarily, collaboration between doctors from different medical fields has been conceptualized as “cooperation among peers” (Tousijn 2012, 525). We argue that, firstly, such *medical dominance* promotes epistemic injustices via systematically deflating versus inflating different epistemic agents’ credibility. Immediately, this concerns health professionals such as nurses and social workers whose testimonies and hermeneutical perspectives do not receive the deserved uptake due to their (formally and informally) subordinated status. Secondly, this also promotes epistemic injustice towards patients indirectly via leading to *institutional opacity*. Institutional opacity refers to a situation where an institution (such as a healthcare system) is intransparent to its agents and/or users in a way that makes it difficult to understand “the rules of the game”, i.e., how to communicate in a way that begets one adequate credibility (Carel and Kidd 2021).

Applying the concept of epistemic injustice (Fricker 2007) in this context enables pinpointing how many practices and issues previously described in empirical research about multi-professional teamwork are both ethically and epistemically detrimental. We don’t only describe how some disciplinary or professional perspectives are marginalized, but also discuss the ethically problematic causes and implications of this exclusion. Our aim is to show that thinking about multi-professional teamwork within this framework of epistemic injustice illuminates underlying mechanisms of surface level inefficiencies. A better understanding of the challenges in multi-professional teamwork can hopefully help to target ameliorative measures. At the same time, thinking through the case of multi-professional teamwork can contribute to the philosophical literature on epistemic injustice. Firstly, it strengthens recent applications of the concept of epistemic injustice to institutional actors by demonstrating how epistemic injustice can occur in ways that are irreducible to individual actors. Secondly, while the literature on epistemic injustice in healthcare has been burgeoning, it has to date almost exclusively focused on experiences of such injustices by patients. We add to this literature by looking at the other side of the patient-provider relation, and point out interactions between epistemic injustices on different levels within the healthcare system.

The paper will proceed as follows. Sect. ”Complex teams, disciplinary frameworks, and medical dominance” will go deeper into the cultural and disciplinary differences involved in multi-professional work and take a closer look at the phenomenon of medical dominance. Sect. ”Pathocentric and institutionalized epistemic injustice” will give a brief summary of previous work on (pathocentric) epistemic injustice and relate our argument to recent discussions of institutional epistemic injustice. Sect. ”Epistemic injustice & multi-professional teams” will apply this discussion to the case of medical dominance in multi-professional teams and explicate how this leads to epistemic injustice towards health professionals as well as patients. We finish with a brief conclusion on potential ameliorative measures.

## Complex teams, disciplinary frameworks, and medical dominance

Multi-professional teams are in contrast to the traditional, speciality-focused model of healthcare. In the traditional model, care is organized around individual disease categories, exemplified by clinics specialized in treating different diseases, and social care (e.g., housing services) being separated from clinical care (e.g., Lynch et al. 2022). In recent years, this model has been questioned, as patients with complex problems or multiple diagnoses have faced challenges in navigating the siloed system. Multi-professional teams consisting of social workers, nurses, medical doctors and other professionals are hoped to improve the efficiency and quality of care of these patients. The aim of these teams is to work together towards a common goal. For example, they make decisions about discharging geriatric patients, organizing treatment and rehabilitation of individuals with substance use issues, and improving the quality of life of patients in palliative care (Ellis and Sevdalis 2019; Ullrich et al. 2023). However, as empirical studies have shown, there are challenges to collaboration across professional lines (e.g., Colombo et al. 2003; Tousijn 2012; Maddock 2015; Ellis and Sevdalis 2019). In this paper, we build on this existing literature and connect it to philosophical accounts of interdisciplinarity and epistemic (in)justice. In particular, we focus on challenges that are related to overcoming differences in epistemological, conceptual, and ethical disciplinary frameworks.

In previous work in philosophy of science, conceptual, epistemological, and methodological differences have been identified as obstacles to interdisciplinary collaboration (MacLeod 2018). While 'domain specificity', i.e., the ability to perceive and solve problems on the basis of a specialized set of conceptual tools, epistemic principles, and material or technological resources, helps researchers target complex problems in their own field effectively, it can hinder collaboration across disciplinary boundaries. This is because concepts and principles can be difficult to translate from one domain to another (MacLeod 2018; see also Longino 2002 on local epistemologies). The skills and knowledge an expert needs to have in order to successfully operate in their field may not be clear to outsiders, which makes understanding decision-making in the field difficult. MacLeod (2018, p 707) has called this 'the opacity of domain specific practices'. Successful collaboration then requires that experts are willing to try to understand the disciplinary perspectives of other members of the research team and aim at integrating different disciplinary perspectives (van Baalen and Boon 2024). Moreover, epistemic humility (Ho 2011), i.e. ability to recognize the limits of one’s own disciplinary perspective and willingness to assess the relevance of one’s knowledge to solving the situation at hand, is needed in multidisciplinary decision making. We hold that similar challenges and requirements can be found in the context of multi-professional healthcare.

Domain specificity in healthcare is apparent in the differences that exist between how different professional groups perceive their goals and what theoretical and conceptual assumptions underlie their practice. For instance, it has been argued that due to formalized training, different professional groups have diverting understandings of the etiology and nature of and potential interventions on diseases, as well as the nature of care, which contributes to tensions and communicative challenges within multi-professional teams ( e.g., Barrow et al. 2015). These differences are illustrated by a quote from a social worker who was interviewed by Tousijn (2012, p 532, brackets in the original):

“They [the healthcare professionals] have a healing culture, in which a patient is treated for a while, and then you get a result. We [social workers] are more conscious of the long-term dimension of many problems, in which change is slow.”

An example of disciplinary perspectives that influence multi-professional work are different approaches to mental health. For example, distinct implicit models of schizophrenia have been identified as an interfering factor in the collaboration between psychiatrists, social workers, and community psychiatric nurses (Colombo et al. 2003). While psychiatrists implicitly accept ‘a medical model’ (according to which mental illness is caused by biological factors), social workers mostly support ‘a social model’ (according to which socioeconomic factors, marginalization etc. are causes of mental illnesses) (Colombo et al. 2003, see also Maddock 2015). Consequently, professionals disagree, e.g., on the likely causes of a patient’s condition and how it could be improved (psychiatrists favor medical and surgical interventions, while social workers see change in the social circumstances as an important intervention). Due to these differences, conflicts and misunderstandings arise, making decision-making in a group more difficult. Care of individuals with substance use disorders is another example of a context where disciplinary differences in (implicit) assumptions about the nature of the patients or clients’ problems cause tensions. For instance, professionals who emphasize the biological basis of addiction and those who focus on psychosocial causes of drug use have disagreed on whether resocialization, e.g., change in the social environment of the individual, can be a solution to drug use (e.g., Larsen et al. 2022).

Professional differences can extend beyond concepts, methods, and epistemologies to ethically relevant understandings of one’s own role. For instance, Copeland (2020) points out differences in conceptualizations of pain between medicine and nursing. Whereas many physicians define the experience of pain in relation to (actual or potential) tissue damage, nursing has widely adopted a patient-centric definition of pain, according to which “pain is whatever the experiencing person says it is, existing whenever and wherever the person says it does” (Copeland 2020, p 1). This is related to ethical understandings of the role of nurses; in particular, it is connected to a self-understanding as patient advocates, who help preserve patients’ dignity. Copeland argues that this leads (or should lead) to differences in the description of, and response to, patient requests for pain medication.

Relatedly, the International Code of Ethics for Midwives^2^ describes the relation between a midwife and a patient/client as a partnership, and it considers the empowerment of patients a central obligation. In practice, this can often mean to support gestating subjects in making informed decisions that can go against the dominant view in thoroughly medicalized settings for pregnancy and birth. The ideal midwife would thus not just perform a medical service, but enable their patients’ dignity, autonomy, and safety in situations prone to obstetric violence (i.e., neglect, disrespect, or abuse by health workers; cf. Chadwick 2021; WHO 2014). For instance, Shaw (2013) describes the practice and profession of midwifery in terms of resistance to medicalization and pathologization of normal processes and experiences.

“Midwifery care seeks to honor and empower women to have control over their birthing experiences, thus emancipating themselves from the patronizing care that often exists under the medical model.” (Shaw 2013, 530).

One can easily imagine how such different conceptualizations of birth, and birthing care, can lead to conflicts between different professional groups in clinical practice. In general, as the above examples show, multi-professional teamwork cannot simply be understood as people with different educational backgrounds combining particular pieces of knowledge and skills. Instead, different professional cultures can differ in their epistemological, metaphysical, and conceptual outlooks on the problem at hand (see also van Baalen & Boon 2024). The domain specificity (MacLeod 2018) can lead to conflicts regarding assessments of the best treatment and care. This is because multi-professional teams are not just aggregating information but also need to integrate different paradigms and hermeneutical perspectives. In Kuhnian terms, health care professionals are socialized to be members of particular communities during their specialized education, which includes the development of particular styles of thought and approaches to health problems and patient care. While this may be a necessary feature of training, it does not teach the skills needed for interdisciplinary and -professional communication and collaboration.

Disciplinary differences can be particularly tricky in cases of an individual having more than one diagnosis. An example would be a patient having both a mental health diagnosis and substance use issues. In these cases, challenges are created not only by conceptual and epistemological disciplinary boundaries but also by sectoral boundaries and fragmentation because the treatment of substance abuse and mental health problems is typically organized by different services (Fantuzzi and Medina 2020). Another example are patients with both somatic and psychiatric illnesses, who often suffer from the effects of mental and somatic health care institutions lacking integration (Bueter 2023).

Furthermore, multi-professional teamwork is typically characterized by power asymmetries. Despite ‘partnership’ and ‘collaboration’ and ‘shared leadership’ being emphasized in principle, in practice teams function hierarchically with physicians usually acting as leaders (e.g., Lingard et al. 2012). This reflects what sociologists have named ‘medical dominance’. This concept has been used to refer to the authoritative position that medicine as a profession (and as a field of science) has in the field of healthcare (Willis 1983). The power of medicine is claimed to be apparent on multiple fronts: Not only are medical doctors often able to make decisions about how other professionals carry out their work (see, e.g., Newham 2014 on the relationship between midwives and obstetricians) and how patients should treat their illness, but they also often hold positions of power in healthcare institutions, which gives them administrative authority (e.g., Kenny and Adamson 1992). What is central to the sociological discussion on medical dominance is that the authoritative position of medical doctors and medicine is taken to be constituted by institutional factors. For instance, in many countries medical doctors have the monopoly to diagnose and make referrals, and consequently decide who will receive publicly funded or insurance-covered treatment (Kenny and Adamson 1992; Germov 2019). Similarly, in many jurisdictions only medical doctors, dentists, and veterinarians have the right to prescribe medication (Bourgeault and Mulvale 2006).

Some medical sociologists have argued that medical professionals having administrative, financial, and decision-making authority is related to what the knowledge base guiding healthcare is. For example, according to Germov (2019, p 390), medical professionals have more administrative power in many decision-making bodies (e.g., hospital management boards, boards making decisions about funding health research) than other health professionals. As a consequence, Germov claims, non-medical professionals have less opportunities to decide how to conduct research in non-medical paradigms and generate evidence relevant for developing their practices. Similarly, Colombo et al. (2003) connect the dominance of the medical model of mental health and disease to the authoritative position that psychiatrists have in healthcare.

In the next sections, we build on these descriptions of different professional frameworks and medical dominance. By drawing on the influential work of Miranda Fricker, Havi Carel, and Ian Kidd, we argue that medical dominance in multi-professional teams can lead to an institutional epistemic injustice that affects both healthcare professionals and patients negatively.

## Pathocentric and institutionalized epistemic injustice

Since Miranda Fricker (2007) introduced the concept of epistemic injustice to designate a special form of injustice that undermines someone in their capacity as a knower, a flourishing field of philosophical inquiry has developed. This holds in particular for the inquiry into epistemic injustice in healthcare contexts. Fricker’s original account distinguished two forms of epistemic injustice: testimonial and hermeneutical injustice. *Testimonial injustice* occurs when a hearer assigns a speaker less credibility than they deserve due to negative identity prejudices. This form of epistemic injustice is agential and transactional, i.e., it is typically perpetrated by individuals in particular communicative encounters. As the relevant prejudices often track the speakers in various, if not all, social contexts (i.e., due to widespread gendered or racialized prejudices), testimonial injustice can be very persistent and systematic, its harms thereby cumulating.

*Hermeneutical* injustice occurs where identity prejudices and social oppression cause a linguistic community’s pool of conceptual resources to exhibit a certain kind of gap: a lack of shared concepts to describe the experiences of precisely those people that are excluded from, or at least underrepresented in, meaning-making practices such as journalism, science, or law. Such conceptual gaps make it difficult to understand, as well as to effectively communicate, one’s experiences of, e.g., sexual harassment, hetero- and cis-normativity, or white ignorance. Hermeneutical injustice of this kind is structural. It affects all speakers within a linguistic community equally; e.g., both the queer person and the queerphobic person have difficulties putting the relevant phenomena into words. Yet, the resulting disadvantages and harms are unequally distributed: this situation is obviously harder for the queer person, and may serve the queerphobic one.

Hermeneutical injustice codifies social power and prejudices into our very language by means of hermeneutical marginalization. Mitigation thus calls for structural countermeasures targeting such marginalization. Testimonial justice, too, happens in an environment structured by social power hierarchies (at least in its systematic rather than incidental variant). Working within a virtue-theoretical framework, however, Fricker (2007) mostly focuses on the virtues of testimonial and hermeneutical justice as countermeasures. A person displays testimonial justice when they correct for their own prejudicial credibility assignments; they display hermeneutical justice when they give someone whose articulation of certain experiences seems hard to understand the benefit of the doubt.

The concept of epistemic injustice has been used for analyzing encounters in healthcare. Havi Carel and Ian James Kidd have been seminal in exploring epistemic injustice faced by patients, arguing that ill people are particularly vulnerable to epistemic injustices due to pathophobic prejudices (e.g., Carel and Kidd 2014, 2017; Kidd 2020; Kidd and Carel 2017; Kidd et al. 2023). For instance, people with chronic somatic diseases are often considered as cognitively impaired due to the very illness experience, or people with mental disorders get treated as untrustworthy or generally incapable of rational thinking (e.g., Crichton et al. 2017; Scrutton 2017). The burgeoning field of *pathocentric epistemic injustice* has, moreover, examined experiences of epistemic injustice focusing on particular diagnoses, such as CFS/ME, depression, or dementia (Blease et al. 2017; Jackson 2017; Spencer 2023). Epistemic injustice towards healthcare professionals, in turn, has been discussed, e.g., by Reed and Rishel (2015). According to them, epistemic injustice contributes to communication failures in end-of-life care: nurses are marginalized and their concerns and expressions of moral distress are not taken seriously when decisions about care are made.

These applications to the world of medicine and psychiatry have, in turn, paved the way for systematic developments in the theory of epistemic injustice. For instance, Kidd and Carel (2017) argue that hermeneutical injustice can also take the form of discrediting certain styles of expression and point to structural features of healthcare systems (such as high patient loads and rigorous time-constraints) as promoting testimonial injustice. This aligns with developments in the general debate on epistemic injustice, which call for structural accounts of testimonial as well as hermeneutical injustice–and, consequently, for structural countermeasures (e.g., Anderson 2012; Bueter 2023).^3^

While being careful to avoid an inflation of the concept of epistemic injustice (to keep it sharp and useful), Fricker has also reacted favorably to some accounts that broaden her original framework (e.g., Fricker 2017). Of particular relevance to our purposes here is the recent discussion of *institutional epistemic (in)justice*. If testimonial and hermeneutical (in)justice are conceived of as virtues and vices, the question arises whether we can ascribe them to institutions at all. Fricker (2021) argues that this is not only possible but also desirable, as it makes certain phenomena easier to pinpoint and analyze.^4^ For instance, this can be helpful in situations where institutional structures create conditions that lead to epistemic injustice without the people working for that institution (e.g., the healthcare system or the judicial system) being prejudiced against the victims of said injustices (Fricker 2021, 90; cf. also Fricker 2010).^5^

To achieve this, Fricker introduces the notion of an *institutional ethos* as analogue to an individual’s character. This captures the phenomenon that we do not just wish for institutions that produce the right kinds of outcomes (e.g., adequate sentencing in the judicial system), but that do so for the right reasons or value commitments. For instance, we probably want the healthcare system not only to deliver effective care but, ideally, for this effective care to be based on a value commitment to help alleviate suffering (rather than, e.g., a commitment to earn money via effective care). According to Fricker (2021, 91f),

“[i]nstitutional ethos matters, then, partly because the presence or absence of appropriate value priorities behind any given item or process of institutional epistemic conduct is a factor in determining confidence and satisfaction levels in the outcome judgment itself.”

This characterization of an institutional ethos provides a framework to analyze potential shortcomings in cases of dissatisfaction and loss of trust in healthcare. As Fricker (2023) points out, the value commitments making up an institutional ethos could be problematic, or the institution could diverge systematically from its value commitments. For example, this might happen because divergence from the ethos is insufficiently sanctioned (e.g., disrespectful treatment of patients being tolerated), or because the institution starts to utilize processes and establish standards that conflict with its ethos (e.g., shortening the time span of patient-clinician contact more and more). Therefore, institutions can either be committed to values that directly conflict with epistemic justice (e.g., certain political parties, totalitarian regimes, etc.), or they can employ structures and processes that allow for epistemic injustice or even make it a prescriptive norm. As an example of the latter, Fricker (2023) argues that the U.S. policing system is a testimonially unjust institution, because it assigns a problematic credibility excess to incriminating testimonies and confessions (no matter how they have been elicited), whereas retractions of such confessions suffer from credibility deflation.

Carel and Kidd (2021) also develop an account of institutional testimonial (in)justice, building onto Fricker’s notion of an institutional ethos as the “collective motivational dispositions and evaluative attitudes” orienting that institution’s activities (Fricker 2021, 90). They further distinguish between the values, procedures, and outcomes of an institutional ethos, which need to be properly aligned. Institutions such as the judicial or the healthcare system have an ethos of testimonial justice; i.e., a commitment to adequate, non-prejudical credibility assessments (as well as matching procedures and outcomes). Such an ethos, they argue, can degrade if an institution becomes intransparent, or *opaque,* in a way that impedes epistemic agency:

“Institutional opacity is a general tendency within large-scale and internally complex institutions to increasingly become resistant to forms of assessment and understanding. Institutions may be opaque to their *agents*, that is, those who work in them as teachers, nurses, police officers, and so on; they may also be opaque to their *users,* the people who engage with the institution to access services like healthcare or education, or to obey legal requirements, like a court summon or compulsory education.” (Carel and Kidd 2021, 481).

Institutional opacity can make it very challenging to navigate a system, because it will be hard to understand whom to address which questions to, what the right order of steps is, what kind of behavior is considered adequate, and so on. The opacity extends to the relevant economies of credibility, making it difficult for people to act in ways that will gain them credibility (i.e., via employing the right terminology and displaying the right demeanor in a variety of situations), which can undermine epistemic confidence as well as trust in the institution (Carel and Kidd 2021, 482f.). One is, so to say, forced to participate in a game without knowing its rules (but with often very high stakes). Carel and Kidd (2021) illustrate the notion of institutional opacity (and its detrimental outcomes) by the example of the NHS, which officially commits to values such as dignity of patients but diverges from this in its procedures and outcomes. They moreover argue that institutional opacity is particularly harmful to people who are already in epistemically vulnerable positions (as many patients are).

This previous work on institutional testimonial injustice has established the possibility to consider institutions themselves as epistemically (un)just. To summarize:

An institution is epistemically unjust if

1. its institutional ethos is characterized by value commitments to epistemic inequity, and/or
2. it diverges from a given ethos of epistemic justice in its procedures and outcomes; e.g.,
  - by creating formal hierarchies that codify an unwarranted priority of certain perspectives, thereby leading to a systematic inflation vs. deflation of different actors’ credibility.
    (i) Such unwarranted hierarchies of credibility can lead to epistemic injustice even in the absence of prejudice,
    (ii) or they can align with and reinforce existing prejudices against members of particular groups.
  - by creating hierarchies of credibility that are intransparent to agents and users, i.e., institutional opacity.
    (i) Again, institutional opacity can lead to epistemic injustice in the absence of prejudice,
    (ii) or it can align with and reinforce existing prejudices.

In the following, we will examine how this applies to multi-professional healthcare teams. We will start by demonstrating that multi-professional teams are committed to an ethos of epistemic justice (Sect. ”The institutional ethos of multi-professional teams”). We then argue that medical dominance is institutionally unjust because it conflicts with this ethos. Medical dominance implies structuring multi-professional teams in an epistemically inequitable way that codifies an unwarranted, general credibility inflation versus deflation of different professional perspectives (Sect. ”Epistemic injustice within multi-professional teams”). In addition, we will explicate that this has implications for the users, i.e., the patients treated by multi-professional teams, because epistemic inequity on the inside will tend to produce institutional opacity on the outside (Sect. ”Multi-professional teams and institutional opacity”).

## Epistemic injustice & multi-professional teams

### The institutional ethos of multi-professional teams

Large-scale healthcare systems such as the NHS can be ascribed an institutional ethos of testimonial justice, since they are explicitly committed to knowledge-based and respectful treatment of patients. Such an overall commitment implies the need for adequate credibility ascriptions to patients, because dismissing their perspectives and testimonies leads to epistemic losses and suboptimal care. Smaller institutional bodies within these systems likewise share an ethos of epistemic justice. Regarding the case of multi-professional teams in healthcare, an explicit motivation for such teamwork is the aim of better care based on the sharing of knowledge from multiple perspectives (e.g., Babiker et al. 2014). This can only be successful if none of the relevant perspectives get systematically and unfairly deflated.

An explication of this institutional ethos can be found, for instance, in a discussion paper on “Core principles and values of effective team-based health care” by the Institute of Medicine (Mitchell et al. 2012). For example, this document lists “humility” as one of the core value commitments of multi-professional teamwork:

**“Humility:** Team members recognize differences in training but do not believe that one type of training or perspective is uniformly superior to the training of others. They also recognize that they are human and will make mistakes. Hence, a key value of working in a team is that fellow team members can rely on each other to help recognize and avert failures, regardless of where they are in the hierarchy.” (Mitchell et al. 2012, 5).

In other words, a commitment to epistemic humility is made in the document. Vis a vis the phenomenon of medical dominance, this holds that credibility ascriptions should be determined by the respective relevance of one’s training and perspective, rather than by whether one is, e.g., a physician or a nurse. This relevance is determined by the particular task and situation at hand. While not everyone’s expertise applies to all cases in the same way, it is expressed that the team should operate against a background assumption of general epistemic equality, where everyone can contribute or err just the same.

At the same time, this does not frame the existence of a hierarchy in healthcare teams as problematic per se. As the aim here is knowledge sharing and creation for a practical and highly time-sensitive purpose (helping patients), there is no room for endless discussions resulting in a pluralist agreement to disagree. It will be necessary to make decisions and to ensure accountability.^6^ Another core principle listed is therefore a clear distribution of roles:

“**Clear roles:** There are clear expectations for each team member’s functions, responsibilities, and accountabilities, which optimize the team’s efficiency and often make it possible for the team to take advantage of division of labor, thereby accomplishing more than the sum of its parts.” (Mitchell et al. 2012, p 9).

However, just as it is not assumed that everyone will have the same degree of knowledge about everything, or that one profession always knows best, it is pointed out as important that the hierarchy should be structured according to specific tasks and competencies, rather than professional status: The discussion paper states that “effective teams require a clear leader, and these teams recognize that leadership of a team in any particular task should be determined by the needs of the team and not by traditional hierarchy” (Mitchell et al. 2012, p 12). From this perspective, then, the problem with medical dominance is not the existence of a hierarchy as such, which is considered necessary for effective decision-making and accountability. Rather, what is problematic is assigning physician’s perspective higher relevance and credibility across the board.

We take this to express a commitment not to an overall equality of credibility of team members, but to adequate and task-/domain-specific credibility ascriptions. This reminds of Helen Longino’s condition of “tempered equality of intellectual authority” for social objectivity in scientific research (Longino 1990).^7^ That condition also does not require that everyone has the same level of intellectual authority, but that differences in intellectual authority are based on competence, not social-demographic factors such as race or gender. Applying this to our case, differences should not be based on professional status in such a way that it is assumed that “the doctor always knows best”, no matter the concrete situation and task. The idea is to give credibility to the degree it is deserved. While it is assumed that there is a general epistemic equality in the sense that different perspectives can each be valuable (or limited) and everyone can err (regardless of professional background), it is also acknowledged that different perspectives and competencies can be more or less relevant and helpful to particular areas, cases, or tasks. While all team members are thus ascribed the same general level of competence as knowers, the relevance of their knowledge is situation-specific. For instance, social workers can be assumed to have expert knowledge of what services are available for outpatients, which makes their opinion particularly relevant when decisions about discharging patients are made. The credibility assigned to testimonies of professionals may thus legitimately differ in certain situations. In fact, this is the raison-d’étre of multi-professional teams: only if different perspectives are all valuable, but also all limited, does it make sense to combine them.

We will refer to this as a commitment to *epistemic equity* in the following. Epistemic equity expresses a view of various kinds of professional expertise as having strengths and limitations in different regards, therefore ideally complementing each other. Moreover, it assumes that all knowers are basically created equal, in so far as everyone can err. While a principle of epistemic equity amounts to an evaluative stance towards different kinds of knowledge bodies and traditions, it arguably also grounds a richer normative stance of epistemic justice. If certain perspectives are receiving an unfairly low degree of credibility due to prejudice, this will interfere with successful multi-professional teamwork. Moreover, framing the issue in terms of values (or virtues) such as humility (alongside, e.g., honesty or curiosity; Mitchell et al. 2012, 5) indicates a commitment to epistemic injustice not only for prudential reasons, but also as a matter of respect among team members. For instance, the Institute of Medicine discussion paper also lists “mutual trust” as a foundational principle of successful teamwork, pointing out that this requires that all voices are heard, and that team members have good personal relationships (Mitchell et al. 2012, 14ff.).

Multi-professional healthcare teams, in summary, have an *ethos of epistemic justice,* which is based on an assumption of epistemic equity. This includes the principle of testimonial justice, i.e., the credibility of someone’s testimony should not be generally inflated or deflated simply because of their professional background. Moreover, it extends to hermeneutical justice. As has been described above, one of the challenges in multi-professional teamwork is that professional differences cannot be reduced to variety in domain-specific information, but can involve different metaphysical background assumptions, methodologies, terminologies, or ethical orientations and role conceptions. This leads to different hermeneutical perspectives on patients' illness experiences within a team. These different hermeneutical perspectives then would likewise need to be treated equitably.

### Epistemic injustice within multi-professional teams

The ethos of multi-professional teams is characterized by an assumption of epistemic equity and, based on that, a commitment to epistemic justice, i.e., to assigning an amount of credibility to different perspectives and testimonies that matches their relevance and adequacy in a given situation. However, this does not imply that multi-professional teams are in fact epistemically just. As pointed out by Fricker as well as Carel and Kidd, an institution committed to epistemic justice in its value-orientation can still fail to live up to this commitment due to problems with procedures and outcomes (see Sect. ”Pathocentric and institutionalized epistemic injustice”).

Consider the following scenario. A woman is in the process of giving birth at a hospital, accompanied by a midwife to whom she has already formed a relation during prenatal care. The midwife and the obstetrician on service that day disagree on the necessity of an episiotomy. Episiotomies are a standard practice during births at the hospital (as they used to be, because natural tears were thought to be more problematic). The midwife points out that new research indicates that the harm-benefit ratio for episiotomies is far worse than previously thought, and that she has experienced many women having long and difficult recoveries and increased postpartum pain after episiotomies. In general, the midwife adheres to a perspective on birth as a healthy and natural process, whereas the obstetrician focuses on the medical control of this process, believing it to be safer for mother and child. In addition, this decision needs to be made in a relatively short time-frame and the woman in labour is in a high-stress situation. She would like to avoid an episiotomy if possible, but also wants to do whatever is best for her baby.

In this situation, epistemic injustice could arise in a variety of ways. It might show up in a classic, agential and transactional style: The physician does not pay much attention to the midwife’s concerns, because of a prejudice that midwives lack the qualifications for understanding medical research and making medical decisions. They also consider the argument from professional experience as irrelevant. The midwife’s testimony is assigned less credibility than it deserves due to an overly negative view of this profession (and maybe even the women practicing it^8^). This could be countered by a virtue of testimonial justice, which would lead the physician to at least check the possibility that the midwife is right.

But testimonial injustice can also occur here even if the physician is not prejudiced against midwifery and/or midwives. Let’s say the hospital has a commitment to multi-professional maternity and intrapartum care. However, the obstetrician and the midwife don’t know each other yet, and hospital procedures are structured in a way that enforces medical dominance. There is no model for dealing with disagreements other than the physician making the choice and, ultimately, being liable for it. The midwife’s credibility is not deflated by the physician, but the higher credibility of physicians’ testimonies and perspectives is built into the system. In such a situation, it would take a very high credibility assessment of the midwife for the physician to make it seem reasonable to change their position and diverge from the standard practice, as it is very risky from their perspective. After all, if the midwife is wrong, any negative consequences would be treated as a fault of the physician.

This hypothetical case illustrates the possibility of institutional epistemic injustice, which can occur in the absence of prejudice and epistemically unjust agents within an institution, even if the institution has a general value commitment to epistemic equity and justice. The institution itself is structured in a way that codifies a credibility deflation for some professions and an inflation for others. The commitment to epistemic injustice in multi-professional teams is undermined by procedures that formalize an epistemic inequity between different perspectives. The outcome is an epistemic injustice towards professionals such as the midwife in our example, even though the concerned physicians may be non-prejudiced and non-culpable.^9^ Accordingly, such cases of epistemic injustice cannot be (fully) mitigated by individual virtues. Instead, the hospital will need to rethink models for collaboration and liability in its teams.

Empirical research on multi-professional teamwork shows that this is not just a hypothetical issue. For instance, Maddock’s (2015, p 246) case study of an Irish multi-disciplinary mental health care team demonstrated how the psychiatrist dominated decision-making about patient management despite the local policies emphasizing “full team participation in order to facilitate holistic bio-psycho-social discussions on patient assessment and care planning”. Similarly, Robinson and Cottrel (2005) report of social workers in multi-professional teams being marginalized due to other members lacking understanding of their profession and its role in healthcare (cf. MacLeod 2018 on opacity of domain specific practices and Ho 2011 on epistemic humility). If the testimony of a team member is disregarded not only due to hierarchy but prejudices and biases related to their profession, it is possible to label this as epistemic injustice (see Sukhera et al. 2022 for a review of implicit biases in interprofessional teams). Medical dominance in multi-professional teams thus diverges from an institutional ethos of epistemic injustice by procedurally codifying epistemic inequity and, potentially, reenforcing prejudiced credibility inflations. The outcome will be experiences of epistemic injustices by healthcare professionals.

### Multi-professional teams and institutional opacity

The ultimate rationale behind multi-professional teamwork in healthcare is to improve the care for patients by allowing for knowledge amalgamation and attendance to various patient needs. As noted above, such treatment can create additional burdens for patients where the multi-professional team conflicts with the continuity of care; i.e., when patients have to interact with various, changing healthcare professionals. If these contact persons come from different professional backgrounds that have different hermeneutic perspectives on the relevant phenomena, and employ different terminologies, patients can experience what we call *hermeneutic confusion*. Hermeneutic confusion needs to be mitigated in a way that enables patients to navigate the conceptual space. Such a mitigation requires a pluralistic orientation, epistemic humility, and epistemic equity among the various health professionals within their care teams.

As an example, imagine a patient with a substance-abuse disorder and a diagnosis of depression, who has regular contact with a general practitioner, a psychiatrist, various nurses, a social worker (and maybe also members of a self-help group). These professionals (and peers) present them with different perspectives on what addiction and depression are, where they stem from, how they interact, and how to treat them. In general, having access to a variety of perspectives does not have to be bad but could be viewed as a fruitful pluralism (e.g., Longino 2002). After all, one perspective may resonate with a particular patient more than another. For instance, some patients may find it helpful to think about their addiction and depression primarily as brain diseases, as this helps alleviate feelings of shame. Others, however, might find the idea of a brain pathology disempowering and hopeless. Multi-professional teams that include people with different disciplinary perspectives can thus have the added benefit of increasing the chance that patients find a framework that resonates with them. Such a pluralism, however, requires disciplinary differences to be communicated effectively; i.e., the relation and status of the differing perspectives should be made clear. Otherwise, patients end up having to navigate these differences without being handed a map, metaphorically speaking.^10^

Turning hermeneutic confusion into a helpful pluralism of perspectives in turn requires that the multi-professional teams live up to the institutional ethos of epistemic justice described above. This is so because epistemic inequity within healthcare teams increases institutional opacity for patients: If different professionals have differing hermeneutical perspectives, and some of these perspectives have more credibility than others due to their proponents’ status in an institutional hierarchy, it will create a situation where patients not only have to deal with hermeneutical confusion/plurality but also with varying norms for expressing themselves and addressing professionals. Their own credibility might then be deflated because the perspective and language most helpful to them is discredited in a system structured by medical dominance.

As an example, take the patient that finds the perspective of their social worker on addiction most helpful. Viewing their substance use in the context of their life history and social circumstances allows them to let go of the belief that they are doomed to continue using. It motivates them to work on their living situation and enables them to believe in their own abilities, including the ability to overcome their substance abuse. If the patient’s health care team is epistemically inequitable in a way that aligns with medical dominance (e.g., prioritizing a medical model of mental disorder), this may lead to experiences of epistemic injustice when talking to members of the team other than the social worker. The patient’s preferred perspective makes them frame and express their issues in a way that deflates their credibility in the eyes of some (but not all) team members.

In consequence, institutions that are characterized by epistemic inequity on the inside will tend to create institutional opacity on the outside, as this creates the complex challenge of fitting one’s testimony, language, and even expressive styles to match the credibility norms of different professions, and to adjust to a professional hierarchy in case of conflicts. The subtleties in terminology and descriptions, which may signify adherence to a particular perspective to the health professional, might however be difficult to decode for the outsider. Multi-professional teams will therefore promote epistemic injustices where they require the patient to play by various sets of rules without full insight into these rules. Such a situation will be created by treatment in multi-professional teams that are officially committed to an ethos of epistemic justice, but are at the same time characterized by medical dominance, i.e., institutional structures and decision-making processes that diverge from the institutional ethos.

## Conclusion

Multi-professional teams play a pivotal role in addressing care needs of patients with complex health issues. Yet, they can only play this role successfully if there is effective communication and coordination between the members of the team and between the team and the patients. One barrier to this are differences in professional cultures and paradigms: as philosophers interested in interdisciplinary research projects have previously argued, different disciplinary perspectives and frameworks can hinder teamwork (MacLeod 2018; van Baalen and Boon 2024). When the principles and practices of other fields are not properly understood by some members of the team, or when professionals are not aware of the boundaries of their own disciplinary expertise, shared decision-making suffers. In this paper, we have argued that in so far as these differences in professional cultures and practices are coupled with intra-professional asymmetries in power, especially medical dominance, they can institutionalize an epistemic inequity that will promote epistemic injustice. First, they may lead to epistemic injustice towards health professionals via credibility deflation. Second, they can result in injustice towards patients via institutional opacity.

It is worth pointing out that we do not argue against the existence of hierarchies, even hierarchies with physicians on top, in general. For instance, medical care in emergencies commonly requires fast decision-making, which depends on a clear distribution of roles and chain of commands as well as medical expertise. However, a significant and growing part of the healthcare system is dealing with patients suffering from multiple and long-lasting issues that are not usefully reduced to biomedical pathologies only. Instead, they require a multi-level management by teams of professionals with different skills that ideally complement each other. We argue that in these situations, a general priority of physicians’ perspectives can be unwarranted, leading to epistemic injustices towards other professionals and, potentially, to worse outcomes for patients.

To conclude this paper, we provide a couple of suggestions for how some of the identified challenges could be overcome or ameliorated. First, reducing the intransparency of domain specific practices is needed for protecting the ethos of epistemic justice, i.e., enabling adequate and task-/domain-specific credibility ascriptions. Members of the multi-professional teams should be able to reflect upon how their own professional perspective affects how they approach the problem at hand and explicate it to other members of the team (cf. van Baalen and Boon 2024; Ho 2011). In addition to being able to express their own perspective, team members should be open to consider the perspectives of others and be more aware of disciplinary differences in how care/cure is perceived. If professionals had a basic understanding of the principles of other fields represented in their team, they could better understand the rationales behind their colleagues’ decision-making (e.g., MacLeod 2018). In practice this could be done by including interdisciplinary or interprofessional courses in the medicine, nursing, etc. curricula (e.g., Mirhabai et al. 2024). This could also reduce the impact that stereotypes about other professions (e.g., Sukhera et al. 2022) have on multi-professional communication and, thus, promote epistemic justice. Moreover, it is important to realize that team-building requires time and effort (Mitchell et al. 2012). Outcomes will be greatly facilitated if team members can work together in relatively stable constellations, get to know each other and establish relationships of trust. Thus, healthcare institutions that work with multi-professional teams need to invest resources into measures such as allocating sufficient time to regular team meetings, proceed with care in recruiting team members, provide help to deal with conflicts, etc..

As pointed out above, institutions can become epistemically unjust despite a value commitment to the contrary if their standards and procedures systematically diverge from this commitment. For instance, this might happen due to large-scale factors such as cost-efficiency trumping all other value commitments, leading to diminished resources allocated to support successful teamwork, insufficient contact time with patients, or unmanageable workloads. With regards to multi-professional teams in particular, rigid hierarchies that are in general led by physicians institutionalize medical dominance and problematic, generalized credibility assessments. Instead, epistemic justice among healthcare professionals will be promoted by more fluid, task-based hierarchies. At the same time, roles, responsibilities, and liabilities need to be clearly distributed and communicated.

For promoting epistemic justice towards patients, it is important to secure continuity of care and minimize hermeneutic confusion as well as institutional opacity. Appointing a contact person as the link between a patient and the multi-professional team could be a means for these purposes. The contact person should be able to help the patient navigate the system and communicate clearly about different options. In particular, they should help the patient understand the rationales behind the made decisions. This requires that the contact person themselves has an understanding that a problem can be approached from multiple perspectives and is aware of the potential differences between the perspectives in the group. Moreover, they have to be able to translate the perspectives to the patient in an understandable way. Also here interprofessional education and training can have a central role.

Interpersonal and institutional epistemic (in)justice are related in complex and often mutually reenforcing ways. At the very least, problematic hierarchies of credibility that are codified into institutional structures can promote prejudicial treatment of others’ testimonies and hermeneutical perspectives. Yet, institutional structures can also lead to epistemic injustice in the absence of such prejudices. Arguing for institutional epistemic injustice is not supposed to diminish the relevance of interpersonal, transactional epistemic injustices; rather, it is intended to complete the picture and thereby further our understanding of epistemic injustice. Similarly, focusing on epistemic injustice towards healthcare professionals seems an important addition to the literature on pathocentric epistemic injustice emphasizing patient experiences. This is important not least because different mechanisms and areas of epistemic injustice will interact in complex systems such as healthcare; for example, in the case of epistemic inequity on the inside of teams leading to institutional opacity on the outside. To conclude, epistemically just institutions may not always be sufficient to avoid interpersonal epistemic injustice, but they are a necessary precondition.

## Footnotes

1 Haggerty et al. (2003) refer to these different dimensions as *relational, informational,* and *management continuity.*

2 https://internationalmidwives.org/resources/international-code-of-ethics-for-midwives/#:~:text=This%20code%20acknowledges%20women%20as,of%20all%20members%20of%20society.

3 In addition, Kidd and Carel (2017) point out the relevance of adequate attention to lived illness experiences and propose a “phenomenological toolkit”, which might help health professionals to develop the virtues of epistemic justice (given working conditions that allow for this in the first place).

4 Fricker does not argue, however, that the vocabulary of virtues and vices is necessary to capture the respective phenomena. Fricker (2023) also notes that one might talk about institutional epistemic dysfunction instead, if preferred.

5 Accounts that conceptualize epistemic injustices as caused by institutional structures or practices independently of the individuals who work in those institutions give rise to the question whether responsibility for an injustice can be attributed to an institution. Assigning moral responsibility to collective agents, e.g., companies or other organizations, is an issue that has been debated by, e.g., Miller (2020). For an account of collective moral responsibility in the context of healthcare, see Müller et al. (2021) and Jukola and Gadebusch Bondio (2023).

6 Multi-professional teamwork poses interesting questions about individual, collective, and institutional responsibility. For reasons of space, we cannot discuss this here, but the idea of an epistemically just collaborative team can create puzzles about who will be accountable in case of medical errors. If a feature of medical dominance is that it will usually be the physician within a team who is legally liable, it can be expected that this physician will give greater weight to their own assessment in decision situations. For instance, Smith et al. (2009) asked different professionals about barriers to collaboration in maternity care. While midwives named differences in philosophies of care as the main factor, obstetricians focused on liability and insurance issues.

7 Longino (1990; 2002) offers four criteria that can be used for assessing the objectivity of research communities. The criterion “tempered equality of intellectual authority” is meant to ensure that the community is open for different points of view.

8 As an anonymous reviewer pointed out, stereotypes about professions are often gendered, or related to stereotypes concerning class, race, or other social factors. For instance, nursing (or midwifery) is viewed as female (e.g., Teresa-Morales et al. 2022). Examining how existing epistemic injustice towards professional groups are connected to societal and historical factors related to development of those professions would be an interesting line of future research, but unfortunately we cannot undertake this task in this paper.

9 It is important to note that individuals are also shaped by the structures surrounding them. Institutional epistemic injustice can promote agential epistemic injustice by reinforcing negative stereotypes. For example, physicians who are always making the last call may develop a belief that this is so because of the lack of knowledge and skills within other professions.

10 In addition, this may create the impression that the various professionals disagree with each other, which can be taken as a sign that they don’t know what they are talking about. This will likely erode trust in said professionals (either all or just some of them).
